# Accuracy Screening for ST Elevation Myocardial Infarction in a Task-switching Simulation

**DOI:** 10.5811/westjem.2018.10.39962

**Published:** 2018-11-30

**Authors:** William E. Soares, Lori L. Price, Brendan Prast, Elizabeth Tarbox, Timothy J. Mader, Rebecca Blanchard

**Affiliations:** * University of Massachusetts Medical School-Baystate, Department of Emergency Medicine, Springfield, Massachusetts; † Clinical and Translational Science Institute, Tufts Medical Center and Institute for Clinical Research and Health Policy Studies, Tufts Medical Center Boston, Massachusetts; ‡ University of Massachusetts Medical School-Baystate, Academic Affairs, Springfield Massachusetts

## Abstract

**Introduction:**

Interruptions in the emergency department (ED) are associated with clinical errors, yet are important when providing care to multiple patients. Screening triage electrocardiograms (ECG) for ST-segment elevation myocardial infarction (STEMI) represent a critical interrupting task that emergency physicians (EP) frequently encounter. To address interruptions such as ECG interpretation, many EPs engage in task switching, pausing their primary task to address an interrupting task. The impact of task switching on clinical errors in interpreting screening ECGs for STEMI remains unknown.

**Methods:**

Resident and attending EPs were invited to participate in a crossover simulation trial. Physicians first completed a task-switching simulation in which they viewed patient presentations interrupted by clinical tasks, including screening ECGs requiring immediate interpretation before resuming the patient presentation. Participants then completed an uninterrupted simulation in which patient presentations and clinical tasks were completed sequentially without interruption. The primary outcome was accuracy of ECG interpretation for STEMI during task switching and uninterrupted simulations.

**Results:**

Thirty-five participants completed the study. We found no significant difference in accuracy of ECG interpretation for STEMI (task switching 0.89, uninterrupted 0.91, paired t-test p=0.21). Attending physician status (odds ratio [OR] [2.56], confidence interval [CI] [1.66–3.94], p<0.01) and inferior STEMI (OR [0.08], CI [0.04–0.14], p<0.01) were associated with increased and decreased odds of correct interpretation, respectively. Low self-reported confidence in interpretation was associated with decreased odds of correct interpretation in the task-switching simulation, but not in the uninterrupted simulation (interaction p=0.02).

**Conclusion:**

In our simulation, task switching was not associated with overall accuracy of ECG interpretation for STEMI. However, odds of correct interpretation decreased with inferior STEMI ECGs and when participants self-reported low confidence when interrupted. Our study highlights opportunities to improve through focused ECG training, as well as self-identification of “high-risk” screening ECGs prone to error during interrupted clinical workflow.

## INTRODUCTION

Interruptions, defined as activities that briefly disrupt a primary task, are frequent in the emergency department (ED). Emergency physicians (EP) are interrupted 5–15 times per hour.^1^,^2^ Interruptions have been associated with increased rates of error in psychology,^3^ aviation,^4^ and tactical decision-making,^5^ and have been implicated as a cause of preventable medical errors.^6^ However, interruptions are also important when caring for multiple patients in the busy environment of the ED. Screening triage electrocardiograms (ECG) for ST-segment elevation myocardial infarction (STEMI) represents a time-sensitive, critical interrupting task that EPs frequently encounter. STEMI is regarded as a medical emergency; delays in diagnosis increase patient morbidity and mortality.^7^,^8^ Guidelines recommend that patients presenting to the ED with chest pain have a screening ECG performed and interpreted by a physician within 10 minutes of arrival, resulting in multiple interruptions every shift devoted to ECG interpretation from often-unknown triage patients.^9^,^10^

To manage the multiple interrupting ECGs per shift, along with other clinical interruptions, physicians often engage in a cognitive process known as task switching.^11^ Task switching involves briefly shifting away from a primary task to address a secondary, or interrupting, task. Once the interruption is addressed, attention is returned to the primary task. According to cognitive theory, task switching exacts a mental cost; each switch places an increased workload on short-term memory, subsequently increasing the likelihood of error. That said, not all task switching incurs the same mental cost. Factors related to the physician (experience, ability to use cognitive shortcuts), the task (difficulty, similarity to other tasks), and the environment alter the mental cost and subsequent probability of error.^12^

Given the complex cognitive processes involved in task switching, research evaluating interrupted clinical workflow and medical errors remains difficult to interpret.^13^ Observational studies involving EP workflow,^14^ order entry,^15^ and pharmacy dispensing^16^ all support associations between interruptions and errors. However, experimental trials in medication ordering,^17^ surgical procedures,^18^ and clinical decision-making^19^ have failed to find an association between interruptions and clinical errors. Further, previous studies have focused only on detecting errors in completing the primary task, ignoring the accuracy of the interrupting stimuli. There is a paucity of literature applicable to the unique environment of the ED, where correct interpretation of the interrupting task may be more important than primary task completion. Our study explores physicians’ accuracy screening triage ECGs for STEMI in an interrupted, task-switching simulation compared to an uninterrupted simulation. We hypothesized that accuracy interpreting ECGs for STEMI would be lower in the task-switching simulation compared to the uninterrupted simulation.

**Population Health Research Capsule**

What do we already know about this issue?

*While interruptions have been associated with medical errors, they also impart critical information, such as screening electrocardiograms (ECGs) in emergency department (ED) patients*.

What was the research question?

*Evaluate physician accuracy interpreting simulated interrupting triage ECGs compared to uninterrupted*.

What was the major finding of the study?

*We found no significant difference in accuracy interpreting interrupting ECGs for ST elevation myocardial infarction compared to uninterrupted*.

How does this improve population health?

*Not all clinical interruptions are associated with medical errors. Addressing specific, high-risk task factors that promote errors may improve care in the busy ED*.

## METHODS

### Participants

Intern, resident and attending physicians from the three-year emergency medicine residency program at Baystate Medical Center (BMC) were invited to participate. BMC is a tertiary care hospital and regional STEMI receiving center in Springfield, Massachusetts, with 115,000 annual visits. There were no exclusion criteria. The BMC’s institutional review board approved this study.

### Design

We created a 2x2 factorial crossover design in which each participant completed two simulations during the study: a task-switching simulation and an uninterrupted simulation. To limit priming bias and discovery of the primary outcome, all participants completed the task-switching simulation first and the uninterrupted simulation second. During each simulation, participants viewed a series of patient presentation videos (primary task) and interpreted a series of screening ECGs for STEMI (secondary task). Participants were randomized to which of two patient presentation videos (A or B) and which group of screening ECGs (1 or 2) they completed in the task-switching and uninterrupted simulations, respectively. We used randomization to control for unmeasured differences in difficulty in each of the tasks following applicable Consolidated Standards of Reporting Trials guidelines.^20^

### Primary Task: Patient Presentation Videos

The primary task assignment was to view patient presentation videos. We created patient presentation videos to mimic listening to a formal oral presentation of a complex ED patient from a medical student. Two patient presentation videos (A and B) were created and reviewed for content and clarity by clinical experts WS and TJM. Each video included four, four-minute fictitious ED patient presentations, each with multiple possible medical diagnoses. To ensure similar difficulty, patient presentation videos were matched by the type of patient (adult, elderly, pediatric, trauma), number of items in the history of present illness, past medical history, medications, allergies, and physical exam.

### Secondary Interrupting Task*:* Screening ECGs for STEMI

The secondary interrupting task was screening ECGs for STEMI. Two clinical stimuli packets (1 and 2) were created, each containing 13 unique ECGs, (five STEMI, four normal ECGs and four ECGs with non-critical findings). ECGs were obtained with permission from the WaveMaven ECG website.^21^ WaveMaven is a database of 473 de-identified, online ECGs with difficulty ratings and diagnoses assigned by board-certified cardiologists using corresponding patient level data, such as coronary catheterization results. ECGs in each clinical stimuli packet were matched on diagnosis and difficulty rating, as provided by WaveMaven. We then piloted the ECG stimuli packets in a cohort of 12 EPs not affiliated with the study to evaluate for concordance of difficulty between tests (Supplemental material). To conceal the primary outcome of interest, we included chest radiographs (CXR) and laboratory values in clinical stimuli packets, resulting in a total of 20 interrupting stimuli in each clinical stimuli packet.

### Simulation

Prior to the start of the simulation, participants were randomized in blocks of four using sequentially numbered, opaque envelopes to which primary task (patient presentation video A or B), and which interrupting task (clinical stimuli 1 or 2) would be completed in the task switching and uninterrupted simulations, respectively. Researchers who led the simulation (WS, BP, ET) were not involved in the creation of randomization envelopes and were blinded to group allocation until the simulation began.

Participants were instructed that the task-switching simulation was meant to mirror ED workflow. Their assignment was to remember details and form a differential diagnosis for each of four medical-student patient presentations. Participants were advised they would be interrupted every minute with a clinical stimulus: live paper copies of ECGs, CXRs, and laboratory values from unknown patients waiting in triage. They were asked to quickly evaluate the interruption for any actionable finding (YES/NO), write their diagnosis, and rate their confidence in interpreting the interruption (Likert: 1=uncertain; 5=certain.) An actionable finding was defined as a discovery that would require the patient to be brought into the ED from triage for further evaluation. If participants asked about additional information regarding the triage patient, they were told it was not known. Video patient presentations temporarily paused during interruptions, allowing 15 seconds for participants to interpret the interruption and record answers.

After the task-switching simulation, participants completed the uninterrupted simulation in which the patient presentation videos were viewed uninterrupted. Immediately following the videos, participants completed the second set of clinical tasks without interruption or time limit.

**Figure 1:**
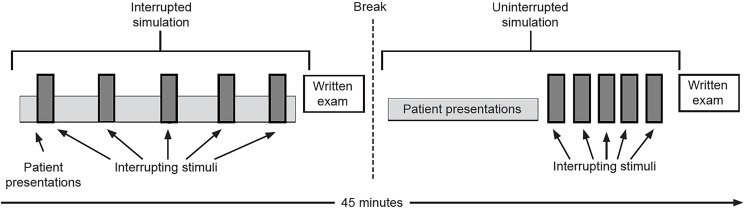
Participant flow through the crossover simulation trial evaluating accuracy of screening electrocardiogram interpretation for ST-elevation myocardial infarction. Participants first completed the task-switching simulation, viewing patient presentations interrupted by clinical stimuli. Then, after completing a written exam and a short break, participants completed the uninterrupted simulation, viewing patient presentations and interpreting clinical stimuli independently and uninterrupted.

At the conclusion of each simulation, participants completed a written exam testing their memory of the patient presentation videos. The assessment included questions regarding details from the chief complaint, past medical history, medications and physical exam of each patient, as well as a final question that asked for a ranked differential diagnoses list for each patient (Figure 1).

### Statistical Analysis

The primary outcome for this study was accuracy interpreting ECGs for STEMI. We defined accuracy as the sum of true positive and true negative interpretations divided by the total number of ECGs. A true positive interpretation was coded if participants correctly identified an actionable finding on the STEMI ECG with a corresponding written diagnosis of STEMI. A true negative interpretation was coded if participants correctly indicated no actionable findings on non-STEMI ECGs, or if participants indicated a finding unrelated to STEMI. We calculated sensitivity and specificity for each participant during each module; we then used means across participants in the final analysis. Paired t-tests were used to evaluate differences in overall ECG accuracy, mean sensitivity and mean specificity in the task-switching and uninterrupted simulations.

To explore associations of accuracy of ECG interpretation for STEMI with clinically relevant covariates, we performed repeated-measures logistic regression using the odds of correct interpretation of each individual ECG as a binary outcome (correct/incorrect). With 26 ECGs for each of the 35 participants, this allowed for up to 910 outcomes. Generalized estimating equations grouped ECGs by participant to account for non-independence of outcomes. Clinically relevant variables defined *a priori* that were incorporated in the model included the following: simulation (task switching, uninterrupted); physician experience (intern, resident, attending); type of ECG (non-STEMI, anterior-lateral STEMI, inferior-posterior STEMI; performance on the primary task (measured by scores on the corresponding written examinations); and confidence in interpretation of interrupting stimuli (dichotomized into low confidence 1–3, high confidence 4–5). To evaluate for effect modification, the interaction of simulation (task switching, uninterrupted) with physician experience, type of ECG and self-reported confidence, were selected a priori for analysis.

### Power

Using paired t-test, with a two-tailed alpha of 0.05, power analysis estimated that 33 participants would allow a power of 0.9 to demonstrate a 0.1 difference in accuracy, or an approximate difference of two ECGs in 26 presented for interpretation, with a standard deviation of 0.20. We performed all statistical analyses using SAS software 9.4 (SAS Institute, Inc., Cary, NC)) and R statistical software (2014. R Foundation for Statistical Computing, Vienna, Austria).

## RESULTS

Thirty-five EPs completed the study and were included in the analysis, including eight of 13 eligible interns, 12 of 24 senior residents and 15 of 28 attending physicians. Years of experience for participants ranged from less than one year to 47 years (median three years, 25^th^,75^th^ percentile= 2,10), whereas nonparticipants ranged from less than one year to 39 years (median three years, 25^th^,75^th^ percentile= 2,19). One participant, a senior resident, completed the simulation but was not included in analysis because his paper data file was lost upon transfer from the simulation center to the secure site.

Participants’ mean accuracy on identification of STEMI by ECG was not significantly different during task switching and uninterrupted simulations (task switching =0.89, standard deviation [SD] = 0.08, uninterrupted=0.91, SD = 0.08, p=0.21). Mean sensitivity of ECG interpretation for STEMI in the task-switching simulation was 0.82 (SD=0.13) compared with mean sensitivity in the uninterrupted simulation of 0.81 (SD = 0.18, p=0.84). Specificity of ECG interpretation for STEMI in the task- switching scenario was 0.90 (SD = 0.19) compared to the uninterrupted scenario of 0.97 (SD=0.07, p=0.07). Stratified by physician experience, there were no significant differences in mean sensitivity or specificity between simulations (Table 1).

Odds of correct ECG interpretation for STEMI were not significantly different between task-switching and uninterrupted groups (odds ratio [OR] [0.81], confidence interval [CI] [0.58–1.12], p=0.32). Covariates related to correct interpretation of ECG included attending physician (OR [2.56], CI [1.66–3.94], p<0.01) and self-reported confidence in ECG interpretation (OR [3.10], CI [2.14–4.50], p<0.01). Presence of an inferior STEMI was associated with decreased odds of correct ECG interpretation (OR [0.08], CI [0.04–0.14], p<0.01). Performance on written exams evaluating memory of the patient presentations were not associated with significant changes in accuracy of ECG interpretation (OR [1.01], CI [0.96–1.05], p=0.82) (Table 2).

**Figure 2:**
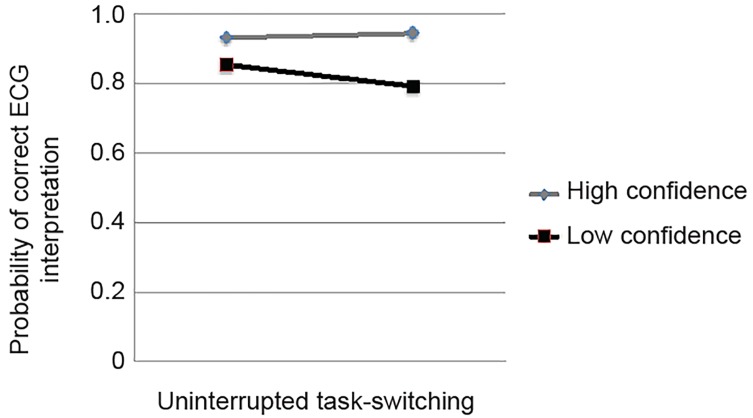
Interaction effect of confidence and scenario on the estimated probability of correct electrocardiogram (ECG) interpretation for ST-elevation myocardial infarction. Low confidence (black) refers to Likert scores of 1–3, whereas high confidence (gray) refers to Likert scores of 4–5. The p value for the interaction was 0.02.

In the task-switching simulation, low self-reported confidence in correct ECG interpretation was associated with lower accuracy of ECG interpretation for STEMI, compared to the uninterrupted simulation (Interaction p=0.02) (Figure 2). Physician experience and ECG-type demonstrated no significant interaction in predicting accuracy of ECG interpretation for STEMI.

## DISCUSSION

The Institute of Medicine’s landmark 1999 report, *To Err Is Human*, highlighted interruptions as a modifiable source of preventable medical errors.^6^ Recommendations have since focused on removing interruptions from clinical workflow.^22^,^23^ However, EPs practice in an environment where interruptions may impart critical information, such as an ECG revealing a STEMI in a triage patient with chest pain. Because we cannot simply remove interruptions from the ED, we must identify and understand modifiable variables that increase the incidence of clinical errors when interrupted.

The aim of the current study was to create a commonly experienced, interrupted-workflow simulation to explore the impact of task switching on accuracy interpreting screening ECGs for STEMI, as well as the variables that influence the likelihood of error. Our study was unique in that it created a scenario that many EPs experience – listening to a medical student’s patient presentation while being interrupted with a clinical task. Further, rather than disregarding the interruption, our study focused on correct interpretation of the clinical interrupting task, a factor uniquely applicable to the EP.

Contrary to our hypothesis, task switching was not associated with accuracy of ECG interpretation for STEMI. There are many reasons why our study may have found no significant difference in accuracy of ECG interpretation for STEMI. First, it is possible that our task-switching simulation was not challenging enough to cause error compared to an uninterrupted simulation. Factors intrinsic to the simulation such as ECG difficulty and time allotted to complete interpretation may have produced less strain on working memory, allowing physicians to switch between tasks without significant differences in measured accuracy.

Yet overall accuracy on ECG interpretation for STEMI was only moderate, with physicians incorrectly identifying between 2–5 of 26 total ECGs. Further, consistent with prior literature we found that physician experience, location of STEMI, and self-reported confidence in ECG interpretation were each associated with improved accuracy, suggesting our ECGs were of comparable difficulty to previous studies.^24^–^30^ Finally, most participants appeared to experience time pressure to complete tasks, as many used all 15 seconds to interpret interrupting stimuli, with some continuing to interpret as the video patient presentations resumed, although this was not formally measured. 31

A theory-based explanation is that task switching has a variable association with errors, changing depending on the mental cost experienced, which is dependent on factors intrinsic to the individual physician and the environment. Previous research suggests that different types of interruptions have different error rates. Interruptions that take more time, use similar cognitive resources, and occur in the middle of the primary task often lead to worse performance.^32^–^34^ Further, physician experience and task difficulty appear to attenuate the mental costs associated with task switching.^35^ Our interrupted-workflow simulation involved EPs evaluating short, clinically relevant, visually based clinical tasks in the setting of visual and auditory patient presentations. It is possible that EPs’ experience and training, both in ECG interpretation and with task switching, resulted in minimal mental costs when engaged in a frequently encountered scenario – screening ECG interpretation for STEMI – leading to no significant differences in accuracy.

This theory is supported by the interaction of simulation with confidence. ECGs that participants reported higher confidence in interpretating demonstrated similar mean accuracies across simulations. Put another way, when interpreting self-reported “easier” ECGs, trained EPs may have experienced minimal mental costs with task switching, resulting in no significant differences. However, for ECG interpretation self-identified as more difficult to interpret, error rates in the task-switching simulation were significantly increased, suggesting increased mental cost for more difficult tasks that may predispose to increased clinical errors when interrupted.

### Future Directions

Our results suggest opportunities for improvement in interpreting screening ECGs for STEMI in a task-switching environment through education in STEMI identification as well as in self-awareness of more difficult-to-complete interrupting tasks. With regard to ECG identification, physicians were significantly less likely to correctly identify inferior and posterior STEMI ECGs in both simulations. Previous literature had demonstrated multiple, subtle STEMI patterns that EPs have higher odds of misclassifying, including posterior STEMI.^36^ Focused training and education on identification of more difficult-to-interpret STEMI ECG patterns may help avoid future errors when physicians are interrupted.

Additionally, although physicians may be poor at global self-assessment,^37^ they may be able to correctly interpret their odds of successful performance on specific clinical tasks. Using complex patient presentations, Friedman et al.^38^ found modest but significant associations of correct diagnoses and level of confidence in students, residents, and attending physicians. Similarly, Eva et al.^39^ demonstrated that psychology students were able to discern trivia questions they were more likely to answer incorrectly, with the study concluding that self-assessment on specific items may be more accurate than global assessments. The ability to apply metacognition and self-assessment in the interruption-driven work environment may have particular relevance to reducing cognitive errors. 40 Future research should focus on evaluating EPs’ ability to self-identify difficult tasks that could increase error in an interrupted environment.

## LIMITATIONS

Our study has many limitations. Most importantly, our results are from a controlled simulation study and should not be over-generalized. Due to standardization and patient safety concerns, prospective simulations could not be performed during actual clinical practice. While we attempted to create an accurate representation of an interrupted, time-pressured ED scenario, many factors cannot be replicated in a simulation. Therefore, our results should be viewed as exploratory and used to highlight factors that can be modified to improve accuracy when interpreting clinical interruptions in the ED.

Additionally, participants were not randomized to the order of the simulation, which may have introduced bias. We chose to standardize simulation order to avoid any effect of task priming, in which participants would have completed multiple ECGs in the uninterrupted simulation immediately prior to the task switching simulation, thus artificially improving their performance. Additionally, because analysis of the primary outcome required that we have a higher proportion of ECGs with STEMI in the simulation, we were concerned that the participants who were randomized to the uninterrupted simulation first would notice the high proportion of STEMI ECGs and subsequently devote unequal attention to the task-switching ECGs, invalidating results. Given that the uninterrupted simulation was completed without task switching or time restraint, we felt that knowledge of the outcome during the uninterrupted simulation would have less of an impact on study validity.

## CONCLUSION

In our simulation, task switching was not associated with accuracy of ECG interpretation for STEMI. However, odds of correct interpretation decreased with inferior STEMI ECGs and when participants self-reported low confidence when interrupted. Our study highlights opportunities to improve through focused ECG training as well as self-identification of “high-risk” screening ECGs prone to error during interrupted clinical workflow.

## Supplementary Material



## Figures and Tables

**Table 1:** Mean accuracy (standard deviation) in interrupted and uninterrupted simulations, overall and stratified by position. Hypothesis testing performed using paired t-tests.

Variable	Task switching	Uninterrupted	P value
All participants (n=35)	0.89 (0.08)	0.91 (0.08)	0.21
Interns (n=8)	0.82 (0.08)	0.88 (0.09)	0.17
Residents (n=12)	0.88 (0.09)	0.88 (0.08)	0.81
Attending physicians (n=15)	0.93 (0.05)	0.94 (0.05)	0.38

**Table 2:** Generalized estimating equations logistic regression (univariate and full model) for factors associated with correct ECG interpretation for STEMI. Low confidence was defined as a self-reported Likert score of 1–3, and high confidence was defined as a self-reported Likert score of 4–5.

	Univariate model			Full model		
		
Variable	OR	CI	P value	OR	CI	P value
Simulation						
Uninterrupted (base)	1.00			1.00		
Task-switching	0.81	0.58–1.12	0.32	0.80	0.51–1.24	0.31
Physician experience						
Intern (base)	1.00			1.00		
Resident	1.30	0.80–2.13	0.26	1.29	0.68–2.47	0.44
Attending*	*2.56	*1.66–3.94	*<0.01	*2.40	*1.42–4.05	*<0.01
Type of ECG						
Normal (base)	1.00			1.00		
Anterior STEMI	1.17	0.44–3.13	0.67	0.78	0.30–2.03	0.61
Inferior STEMI*	*0.08	*0.04–0.14	*<0.01	*0.06	*0.03–0.11	*<0.01
Written exam	1.01	0.96–1.05	0.83	1.01	0.96–1.06	0.62
Confidence						
Low (1–3) (base)	1.00			1.00		
High (4–5)*	*3.10	*2.14–4.50	*<0.01	*3.68	*2.26–6.01	*<0.01

*ECG*, electrocardiogram; *STEMI*, ST-elevation myocardial infarction; *OR*, odds ratio; *CI*, confidence interval.

* p<0.05.
