# Supplementary material for: Accuracy Screening for ST Elevation Myocardial Infarction in a Task-switching Simulation
Source: West J Emerg Med. 2018 Nov 30;20(1):177–84. doi: 10.5811/westjem.2018.10.39962 (PMC6324702; doi:10.5811/westjem.2018.10.39962)
Supplement: Supplementary file 1 [file wjem-20-177-s001.docx]

**Supplemental Material**

**Validation of electrocardiogram (ECG) for clinical stimuli n=12**

To evaluate for concordance of difficulty of clinical stimuli packets prior to the start of the simulation, 12 senior emergency medicine residents and attending physicians outside of BMC and not involved with the study were approached. They were asked to evaluate selected ECG’s for STEMI (yes/no) and rate their confidence for each ECG interpretation (0%-100%).

Mean scores were similar for ST elevation myocardial infarction (STEMI) (Test 1 = 0.87, SD(0.13), Test 2=0.82, SD (0.13)), non STEMI (Test 1 = 0.97, SD(0.08), Test 2=0.96, SD (0.06)) and total (Test 1 = 0.91, SD(0.1), Test 2=0.89, SD (0.08)). Confidence between test was also similar for STEMI (Test 1 = 86%, Test 2=80%), non STEMI (Test 1 = 83%, Test 2=82%), and total (Test 1 = 84%, Test 2=81%). Spearman correlation for total score on ECG test A and B was 0.55.
